# The Dual Targeting of FcRn and FcγRs *via* Monomeric Fc Fragments Results in Strong Inhibition of IgG-Dependent Autoimmune Pathologies

**DOI:** 10.3389/fimmu.2021.728322

**Published:** 2021-08-26

**Authors:** Céline Monnet, Emilie Jacque, Christophe de Romeuf, Alexandre Fontayne, Toufik Abache, Nathalie Fournier, Gilles Dupont, Delphine Derache, Anais Engrand, Aurélie Bauduin, Aurélie Terrier, Alexander Seifert, Cécile Beghin, Alain Longue, Nicholas Masiello, Laetitia Danino, Michel Nogre, Anais Raia, Frederic Dhainaut, Louis Fauconnier, Dieudonnée Togbe, Carmen Reitinger, Falk Nimmerjahn, Wil Stevens, Sami Chtourou, Philippe Mondon

**Affiliations:** ^1^LFB Biotechnologies, Innovation Department, Les Ulis, France; ^2^Artimmune, Orléans, France; ^3^Division of Genetics, Department of Biology, Friedrich-Alexander University Erlangen-Nürnberg, Erlangen, Germany

**Keywords:** FcRn, Fc fragment, Fc engineering, autoantibodies, autoimmune disease, immune complex (IC), Fc gamma receptor (FcγR)

## Abstract

Novel molecules that directly target the neonatal Fc receptor (FcRn) and/or Fc gamma receptors (FcγRs) are emerging as promising treatments for immunoglobulin G (IgG)-dependent autoimmune pathologies. Mutated Fc regions and monoclonal antibodies that target FcRn are currently in clinical development and hold promise for reducing the levels of circulating IgG. Additionally, engineered structures containing multimeric Fc regions allow the dual targeting of FcRn and FcγRs; however, their tolerance needs to first be validated in phase I clinical studies. Here, for the first time, we have developed a modified monomeric recombinant Fc optimized for binding to all FcRns and FcγRs without the drawback of possible tolerance associated with FcγR cross-linking. A rational approach using Fc engineering allowed the selection of LFBD192, an Fc with a combination of six mutations that exhibits improved binding to human FcRn and FcγR as well as mouse FcRn and FcγRIV. The potency of LFBD192 was compared with that of intravenous immunoglobulin (IVIg), an FcRn blocker (Fc-MST-HN), and a trimeric Fc that blocks FcRn and/or immune complex-mediated cell activation through FcγR without triggering an immune reaction in several *in vitro* tests and validated in three mouse models of autoimmune disease.

## Introduction

Immunoglobulin G (IgG)-targeting autoantigens play a central role in several autoimmune disorders, including immune thrombocytopenia (ITP), myasthenia gravis, multiple sclerosis, pemphigus vulgaris, and systemic lupus erythematosus. Strategies such as plasmapheresis, high-dose intravenous immunoglobulin (hdIVIg), and B-cell-depleting therapies (e.g., rituximab) that decrease the levels of circulating antibodies, including autoantibodies, are effective in treating some autoimmune diseases (1). The neonatal Fc receptor FcRn functions mainly in the recovery and recycling of IgG, providing it with a long plasma half-life (approximately 3 weeks) in humans. Several molecules under development, such as modified Fcs and monoclonal antibodies (mAbs), target FcRn to increase endogenous IgG clearance, including that of pathogenic IgG, to levels similar to those seen with plasmapheresis. A modified IgG1-Fc fragment (efgartigimod) encompassing the ABDEG mutations (M252Y/S254T/T256E/H433K/N434F) (2) has shown efficacy in reducing IgG levels in humans (3) and promoted long-lasting disease improvement (4). Several mAbs that block FcRn have also been shown to lower plasma IgG levels in humans (5, 6), with 53.3% of ITP patients showing improvement in platelet counts in a phase 2 study (7). However, currently available FcRn-targeting molecules are likely to be limited to autoimmune diseases in which pathogenic IgGs have a clear correlation with disease activity and severity (8). It is doubtful whether targeting only one receptor subtype will produce sufficiently broad immunomodulatory effects against multifactorial autoimmune diseases (9).

Autoantibodies comprising immune complexes (ICs) induce the cross-linking of activating FcγRs [FcγRI (CD64), FcγRIIIa (CD16a), and FcγRIIa (CD32a)] through their Fc region, which initiates the phosphorylation of immunoreceptor tyrosine-based activation motifs (ITAMs), ultimately leading to the activation of cellular responses such as antibody-dependent cell-mediated cytotoxicity (ADCC) and antibody-dependent cellular phagocytosis (ADCP). The first clinical proof of concept using an Fc fragment to block Fc receptors was reported in 1993 for ITP (10) and Kawasaki disease (11). Additionally, in April 2018, the United States Food and Drug Administration approved the use of fostamatinib (Tavalisse), an oral spleen tyrosine kinase (SYK) inhibitor that blocks the activation of pathways downstream of FcγR (12, 13), as a second-line treatment for patients with chronic ITP. Anti-RhD IgG IC formation with red blood cells also displays efficiency in treating RhD-positive ITP patients through competing with pathogenic ICs. FcγR polymorphisms have also been associated with autoimmune diseases (14). Furthermore, blocking activating FcγRs and modulating their expression represent key mechanisms underlying the ability of IVIg to treat several autoimmune diseases (15).

The FcγRIIIa involvement was clearly shown in humans for the first time in 1986 with the anti-FcγRIIIa 3G8 mAb, which raised the platelet level in 50% of immune thrombocytopenic purpura patients refractory to other treatments (16, 17); however, the 3G8 mAb was found to be toxic. Although this effect was originally explained by its murine origin, a humanized version with deglycosylated Fc was unfortunately also toxic due to the avidity of the mAb leading to FcγRIIIa cross-linking (18–20). Research is now focused on the development of monovalent Fab fragments or short-chain variable fragments (scFv) fused to albumin. scFv-HAS anti-FcγRIIIa murine version and recently a monovalent anti-FcγRIII diabody (3G8 Fab paired with an irrelevant Fab) have demonstrated that previous anti- FcγRIIIa antibodies toxicity may be overcome by monovalent blockade (20, 21). In addition, Norris et al. (20, 21) *in vitro* results indicated the involvement of both FcγRI and FcγRIII but not FcγRIIa in the platelet phagocytosis.

IVIg is considered to be an effective treatment for an increasing number of autoimmune diseases. The major mechanism of action of IVIg seems to be related to its F(ab′)2 and Fc fragments and in particular the dual effect of its Fc region that both blocks activating FcγRs and saturates FcRn (15, 22).

FcRn and FcγRs are currently the targets of emerging molecules that display a dual mechanism of action. Recently, the recombinant multimeric Fc design allowed a higher avidity and an increased binding to FcRn and all FcγRs. Multimeric Fc (stradomers) (23), hexameric IgG1-Fc (24), and trimeric Fc (25) were successfully used to block effector pathways in IC-mediated diseases such as ITP and collagen antibody-induced arthritis (CAIA) in mice. The possible drawback of these Fc multimers is the possibility of FcγR cross-linking, which leads to inflammation. Ortiz et al. (25) demonstrated that constructs containing five or more Fc domains induced FcγR internalization and elicited ITAM signaling. The safety assessments of multimeric Fc molecules are currently in phase I clinical trials.

Using random mutagenesis and phage display selection, we have previously identified several Fc variants of human IgG1 that are optimized for binding to human FcRn. Based on our random mutagenesis approach, we selected key Fc mutations that were unpredictable by rational design and with synergistic effects in modulating the binding not only to FcRn but also other FcγRs (26, 27). Furthermore, the same approach consisting of two rounds of random mutagenesis and selection by phage display relating to FcγRIIIa and C1q has allowed the identification of new key mutations that modulate binding to FcγRIIIa, FcγRIIa, and C1q (unpublished data). Here, we describe the design of a new Fc variant (LFBD192), with six mutations, that displays an improved capacity for binding to Fc receptors (FcRn and FcγRs). This Fc variant was designed using the best variants obtained after random mutagenesis/phage display selection on FcγRIIIa, with the addition of a key mutation that increases its capacity for binding to FcRn. The ability of LFBD192 to block FcRn and IC-mediated cell activation through FcγRs without triggering an immune reaction was compared with that of IVIg, and FcRn blocker (Fc-MST-HN), and a trimeric Fc in several *in vitro* tests. The optimized binding ability of LFBD192 was further validated against the mouse Fc receptors FcRn at pH 6.0 and FcγRIV at pH 7.4. Finally, the efficacy of LFBD192 *in vivo* was assessed in three mouse models of autoimmune disease and compared to that of IVIg, a molecule that is widely used to treat IC-driven inflammatory diseases.

## Materials and Methods

### Construct Production and Purification

Fc-MST-HN and Fc-trimer coding sequences were synthesized according to information available from WO 2016/142782 and WO 2015/168643, respectively. DNA encoding rFc-WT, rFc-C6A-74, rFc-T5A-74, and rFc-A3A-184A was prepared from previously generated constructs using standard PCR procedures (28). The DNA encoding the eight new variants was prepared by overlap extension PCR using primers containing point mutations, with DNA encoding rFc-C6A-74, rFc-T5A-74, or rFc-A3A-184A serving as a template. The PCR products and synthetic gene assemblies were inserted between the NheI and XhoI restriction sites of the OptiHEK vector, a pCEP4 (Invitrogen) derivate. The expression constructs were used to transiently synthesize each molecule in HEK293 cells or FreeStyle 293-F cells (Thermo Fisher Scientific), or expi293 cells (for the Fc-trimer) (Thermo Fisher Scientific) over 6-7 days. Supernatants were subsequently harvested, clarified by centrifugation at 3,000 × *g* for 30 min, and sterile filtered before purification by one-step affinity chromatography using protein A Sepharose 4FF (GE Healthcare, Chicago, IL, USA), elution with 25 mM sodium citrate (pH 3.0), and dialysis in phosphate-buffered saline (PBS). For the Fc-trimer, affinity chromatography was followed by cation exchange chromatography on SP Sepharose (GE Healthcare) to increase the trimeric form ratio of low and high molecular weight species.

The LFBD192 coding sequence was prepared as codon-optimized cDNA for expression in CHO by GeneArt (Thermo Fisher Scientific) and subcloned into the proprietary vector HKgenEFss to improve molecule titer during the bioproduction steps. A stable pool was generated in the Freestyle CHO-S (Gibco, Thermo Fisher Scientific) cell line and used for production in serum-free CD FortiCHO medium containing 8 mM L-glutamine and 4-6 g/L glucose over 10 days in a feedback mode with carbon feed. The supernatant was recovered when cell viability fell below 50%, clarified by depth filtration using a MDOHC filter (Merck), and sterile filtered with a Millipak 200 (Merck) before two-step purification. LFBD192 was affinity-captured with rProtein A matrix (MAbSelect SuRe, GE Healthcare), eluted with glycine buffer (pH 3.0), followed by adjustment to pH 5.5 for HCP flocculation. The filtrated eluate was further purified using a cation exchanger (Capto-S, GE Healthcare). Sodium chloride eluate was concentrated to 20 g/L, formulated by ultrafiltration (Centramate, PALL), and sterile filtered (Sartopore2, Sartorius). Formulated LFBD192 was stored at −80°C. Protein integrity and purity were evaluated by SDS-PAGE, size exclusion chromatography (Superdex 200; GE Healthcare), and dynamic light scattering (Dynapro Nanostar, Wyatt), while endotoxin content was determined by limulus amebocyte lysate (LAL) testing. Protein identity and glycan structure were determined by liquid chromatography-mass spectrometry (LC-MS).

### Octet Binding

Affinities for the human FcRn receptor were determined by Bio-Layer Interferometry (BLI) using a RED96 OCTET system (PALL ForteBio). A recombinant human FcRn receptor (a FcRn α-chain and β2-microglobulin heterodimer) produced in baculovirus (GTP, Toulouse, France) was biotinylated using the EZ-link NHS-PEO kit (Pierce). The biotinylated receptor was diluted to 0.7 μg/mL in phosphate buffer (0.1 M phosphate, 150 mM NaCl, 0.05% Tween 20, pH 6.0) and fixed on streptavidin biosensors (PALL ForteBio 18-5019) for 300 s. Samples were tested for association and dissociation kinetics at 200, 100, 50, 25, 12.5, 6.25, 3.125, and 0 nM in buffer at pH 6.0 for 60 s and 30 s, respectively. Samples were released from the receptor using regeneration buffer (0.1 M phosphate, 150 mM NaCl, 0.05% Tween 20, pH 7.8). For analysis of the results, the biosensors used for the 0 nM measurements served as a reference. The association (Kon) and dissociation (Koff) constants were calculated using a 1/1 model. Recombinant human FcγRIIIa V/CD16aV (#4325-FC, R&D Systems) containing a C-terminal six-His tag was diluted to 1 µg/mL in kinetic buffer and fixed on anti-penta-His (HIS-1K) biosensors for 300 s. Samples were tested for association and dissociation kinetics at 1,000, 500, 250, 125, 62.5, 31.25, 15.625, and 0 nM in kinetic buffer (#18-1092, PALL) for 60 s and 30 s, respectively. Samples were released from the receptor using regeneration buffer (100 mM glycine, pH 1.5). For analysis of the results, the biosensors used for the 0 nM measurements served as a reference. The Kon and Koff were calculated using a 1/1 model.

### Biacore Kinetic Binding Analysis

For FcRn and FcγR kinetics/affinity analysis, a Biacore T200 (GE Healthcare) was used with two different setups, i.e., a sensor chip CAP with a Biotin CAPture Kit (GE Healthcare) was used for FcRn analysis and a sensor chip CM5 with an Anti His Capture Kit (GE Healthcare) was used for FcγR analysis, both following supplier recommendations. Phosphate buffer (20 mM sodium phosphate, 150 mM NaCl, 0.05% Tween 20, pH 6.0) and Tris buffer (15 mM Tris-HCl, 150 mM NaCl, 0.005% Tween 20, pH 7.4) were used as interaction buffers for FcRn and FcγR analysis, respectively. For FcRn analysis, biotinylated human or mouse FcRn (Acrobiosystem, Newark, DE, USA) was reversibly captured (~15–50 resonance units [RUs]) by CAP reagent by injecting 1 µg/mL of the FcRn in phosphate buffer pH 7.4 containing 0.5% BSA at 10 µL/min. Kinetics and affinity measurements were performed by injecting serial dilutions of the sample in single cycle kinetic mode (SCK) at 25°C. For hFcRn analysis, the following concentration ranges were used: 4.7–75 nM for LFBD192 and 24.7–2000 nM for IVIg. For mFcRn analysis, the concentration ranges were 0.2–20 nM for LFBD192 and 3.7–300 nM for IVIg. For FcγRs analysis, His-tagged hFcγRIIIa (Sinobiological) or mFcγRIV (R&D Systems) were reversibly captured (~40–80 RUs) on anti-His antibody by injecting 1 µg/mL of each receptor in Tris buffer at 10 µL/min. Kinetic measurements were performed by injecting serial dilutions of the sample at 25°C in SCK mode for hFcγRIII and multi cycle kinetic (MCK) mode for mFcγRIV. For hFcγRIIIa, the concentration ranges used were 6.25–100 nM for LFBD192 and 31.25–500 nM for IVIg. For mFcγRIV, the concentration ranges were 1.56–400 nM for LFBD192 and 15.6–4,000 nM for IVIg. To correct for non-specific binding and bulk effects, the responses obtained from the reference flow-cell and blank injections were subtracted from each interaction curve. Each sample was analyzed at least three times and mean values are presented. For hFcRn, the pH dependency of association and dissociation were analyzed by injecting a fixed concentration (100 nM) of LFBD192 on 14 RUs of captured hFcRn at different pHs (phosphate buffer at pH 6.0 or 7.4) followed by a dissociation phase in phosphate buffer at the same pH as during injection or applying a pH shift using a dual-injection mode. For the kinetic analysis of the interaction between hFcγRIIIa and LFBD192, a two-state reaction fit was used. To justify the relevance of this type of fit, two experiments were performed. First, to assess whether the interaction involved one or multiple binding sites, a Scatchard plot was generated by injecting increasing concentrations of LFBD192 (6.25–100 nM) in Tris buffer using the MCK mode over 60 RUs of the captured His-tagged hFcγRIIIa. The second experiment was a link reaction test performed using the link reaction wizard of the Biacore T200 control software, i.e., injection of 250 nM LFBD192 at 10 µL/min for a contact time of 0, 5, 3, and 10 min and a dissociation time of 10 min over 60 Rus of the captured His-tagged hFcγRIIIa receptor.

### Competitive Binding Assay *vs* the Fc Receptors Expressed at the Surface of Transfected Cells (FcγRI, FcγRIIa-H, FcγRIIIa-F, and FcRn)

Binding to Fc receptors expressed on the surface of transfected cells (FcγRI, FcγRIIa-H131, FcγRIIIa-F158, and FcRn) was assessed using a competitive binding assay. Binding to FcγRIIIa was performed as follows: In 96-well plates, 2×10^5^ FcγRIIIa-F158-transfected Jurkat cells per well were incubated for 20 min at 4°C with FITC-conjugated anti-FcγRIIIa 3G8 (0.5 μg/mL) and various concentrations (17, 34, 68, 136, 272, 542, 1084, and 2168 nM) of the tested molecules diluted in PBS, pH 7.4. The cells were washed with 100 µL of PBS and centrifuged at 1,700 rpm for 3 min at 4°C. The supernatant was removed and 300 µL of cold PBS was added. FITC-conjugated anti-FcγRIIIa 3G8 residual binding was evaluated by flow cytometry. The mean fluorescence intensity (MFI) values were expressed as percentages, with 100% being the value obtained with the FITC-conjugated anti-FcγRIIIa 3G8 alone and 0% the value obtained in the absence of FITC-conjugated anti-CD16 3G8 (Beckman Coulter). Molecule concentrations required to induce 50% or 25% inhibition were calculated using GraphPad Prism 5 software.

Jurkat cells transfected with FcγRIIa-H or FcγRI were used to evaluate the binding to the respective receptors in the presence of Alexa-labelled rituximab (30 µg/mL). Dilutions were performed in PBS, pH 7.4.

Jurkat cells transfected with FcRn were used to evaluate the binding to FcRn in the presence of Alexa-labelled rituximab (30 µg/mL). Dilutions were performed in PBS, pH 6.0, as binding to FcRn is pH-dependent.

### Inhibition of Anti-D ADCC Against RhD^+^ Red Blood Cells

Peripheral blood mononuclear cells (PBMCs) purified from buffy coats using Ficoll-Paque density gradient centrifugation were incubated at 1×10^6^ cells/well (25 µL of cells at 4×10^7^ cells/mL) with anti-D antibody AD1 (LFB) at 50 ng/mL and RhD^+^ red blood cells (0.5×10^6^ cells/well; 25 µL of cells at 2×10^7^cells/mL) for 16 h at 37°C (an effector/target ratio of 2:1) in the presence of different concentrations (1,500, 150, 15, and 1.5 nM) of the tested molecules. The cytotoxic activity induced by AD1 was measured chromogenically by quantifying the amount of hemoglobin released by the red blood cells in supernatants. The results are expressed as a percentage of specific lysis. The molecule concentrations required to induce 25% inhibition were calculated using GraphPad Prism 5 software.

### Inhibition of Interleukin (IL)-2 Secretion

CD20-expressing Raji target cells (2.5×10^5^ cells/well) were incubated with anti-CD20 mAb (rituximab) at 0.1 µg/mL in the presence of Jurkat cells transfected with FcγRI (1.25×10^5^ cells/well), 10 ng/mL phorbol myristate acetate (PMA), and two concentrations (1,950 and 975 nM) of IVIg or LFBD192. After 16 h at 37°C, the amount of IL-2 released by FcγRI-transfected Jurkat cells was measured by colorimetry (RD System IL-2 DuoSet ELISA Kit, #DY202-05) as described (29).

### C5a Generation

Complement activation in human blood was determined by measuring the C5a concentration. Citrated whole blood (100 µL) was incubated at 37°C in the presence of hirudin (6 µL at 2,000 U/mL), CaCl_2_ (8 µL at 75 mM), and LFBD192, Fc-trimer or IgG at the concentration of 16.5 µM and 33 µM. Lipopolysaccharide (LPS) (83 µg/mL) and heat-aggregated IgG (65°C for 30 min) were used at 2.06, 4.12, 8.25, 16.5, and 33 µM served as positive controls. After overnight incubation, 5 µL of 100 mM EDTA was added to stop the reaction. The plates were centrifuged for 2 min at 1,700 rpm and the amount of C5a in the supernatant was quantified using an ELISA kit (R&D Systems).

### Induction and Activation of Platelet Aggregation

Citrated whole blood was centrifuged at 200 × g for 10 min and platelet-rich plasma (PRP) present in the supernatant was collected. Fc fragments (50 µg; 25 µL at 2 mg/mL) or IgG (150 µg; 25 µL at 6 mg/mL) was added to 25 µL of PRP for 20 min at 37°C. HEPES buffer supplemented with 5% fetal calf serum (FCS) was used as the negative control and TRAP-6 (50 µM final concentration) as the positive control. PRP was diluted (1/100) with HEPES buffer containing 5% FCS. The platelet number was quantified according to the number of CD42b-positive cells and CD62P expression (activation marker) was determined by flow cytometry (FC500 Beckman Coulter).

### Whole-Blood Cytokine Release Assays

Increasing concentrations of LFBD192 or IVIG (6.5, 65, 650, and 6,500 nM) were incubated with citrated whole blood from healthy human volunteers (EFS Nord de France). After 16 h of incubation at 37°C, the blood was centrifuged and the concentrations of cytokines (CXCL10, G-CSF, IL-1β, IL-12/p70, IL-22, IL-5, TNF-α, CXCL9/MIG, IFN-γ, IL-10, IL-1RA, IL-4, and IL-6) released in the supernatants was measured using Magnetic Luminex Assays (human premixed multiple analyte detection kit, #LXSAHM-13).

### Inhibition of Antibody-Mediated Platelet Phagocytosis

Human THP1 monocytes stably transfected with a chimeric molecule consisting of the human FcγRIIIa-V158 extracellular domain joined to the transmembrane and intracellular domains of the gamma chain of the mast/basophil Fc receptor for IgE (THP1 CD16^+^ cell line) were used. The gamma chain allows FcγRIIIa membrane expression and provides the ITAM necessary for the transduction of the phagocytosis signal. THP1 CD16+ cells (1×10^5^) were pre-incubated with increasing concentrations (6.66×10^-5^, 6.66×10^-6^, 6.66×10^-7^, and 6.66×10^-8^ M) of 10% IVIg (Iqymune, LFB) or LFBD192 for 30 min at 37°C. Platelets were isolated from human blood (EFS, Les Ulis), labeled using the PKH67 Green Fluorescent Cell Linker Kit (general cell membrane labeling, Sigma), and sensitized at 37°C for 30 min with stirring with 1 µg/mL of a human IgG1 anti-CD41 monoclonal antibody (Absolute Antibody) or dilution buffer (non-sensitized platelet control). After incubation, samples were washed twice in Iscove’s modified Dulbecco’s medium (IMDM) supplemented with 10% FCS at 1,200 × *g* for 5 min. Labeled platelets (2×10^6^) were added and incubated for 1 at 37°C. PKH67 labeling was quenched by trypan blue and effector cells were labeled with a fluorescent anti-CD45 antibody (Beckman Coulter). Antibody-mediated platelet phagocytosis was measured by flow cytometry (Gallios Flow Cytometer, Beckman Coulter).

### ITP Mouse Model

Humanized FcRn mice (hFcRn; mFcRn^−/−^ hFcRnTg 276 hemizygotes in a B6 background), 12-16 weeks old, were used for all *in vivo* ITP-related experiments. These mice carry a knock-out mutation for the mouse *Fcgrt* gene (Fc receptor, IgG, alpha chain transporter) and carry the human *FCGRT* gene instead, and were obtained by breeding homozygous Tg 276 hFcRn mice with FcRn^−/−^ mice purchased from The Jackson Laboratory. After breeding at Charles River facilities (Lyon), the mice were kept in the animal facilities of Friedrich-Alexander-University Erlangen-Nürnberg under specific-pathogen-free conditions in isolated ventilated cages after at least 5 days of acclimation. The mice were maintained according to the guidelines of the National Institutes of Health and German legal requirements. ITP was induced by intraperitoneal injection of 0.3 µg/g body weight 6A6-hIgG1 antibody as described (15). Platelet counts were determined before and 24 h after the injection of antibody diluted 1:4 in PBS in an Advia 120 hematology system (Bayer). Platelet counts before antibody injection were set to 100%. Mice were pretreated by intraperitoneal injection of PBS (control) and of 50 mg/kg LFBD192, Fc-MST-HN, and the Fc-trimer 2 h before platelet depletion.

### K/BxN-induced Mouse Model of Acute Arthritis

Eight-week-old female C57BL/6 mice were purchased from Janvier (France) and maintained at the Artimmune animal facility before experimentation. To induce arthritis (day 0), one intravenous injection of 10 μL/g K/BxN serum [prepared as previously described (30) was administered. Three days after serum transfer, a single dose of vehicle (PBS), IVIg (2,000 mg/kg), LFBD192 (25, 50, and 100 mg/kg), Fc-MST-HN (100 mg/kg), or Fc-trimer (100 mg/kg) was intraperitoneally administered. Dexamethasone (1 mg/kg) was administered daily and subcutaneously from day 3 until day 9. The therapeutic efficacy of the different molecules was assessed by three different readouts—clinical scores, measurement of IL-6 levels on day 6, and histology (**Figure 7A**). Arthritis severity was scored *via* clinical examination by adding the indices of all the paws: 0, normal paw; 1, swelling of one joint; 2, swelling of more than one joint; and 3, severe swelling in the entire paw (28). At the peak of the disease (day 6), blood was drawn from all the animals and collected into tubes. The tubes were centrifuged at 5,000 rpm for 5 min at 4°C and the serum was collected. Serum cytokine concentrations were determined by multiplex immunoassay (MagPix, Bio-Rad). The results are reported as pg/mL.

The serum concentrations of mouse total IgG were measured by ELISA on days 6 and 10 (Cat-88-50400-22, Thermo Fisher Scientific). A total of 100 µL of coating antibody diluted in PBS was incubated overnight at 4°C. After washing with PBS + 0.05% Tween 20 and then with PBS only, 250 µL of blocking buffer (PBS, 0.1% Tween 20, and 2% BSA) was added to each well followed by incubation for 2 h at room temperature. After washing, 100 µL of standards (two-fold serial dilutions, standard curve range: 1.56–100,000 pg/mL), samples, and blanks were incubated for 2 h at room temperature. After washing, 50 µL of detection antibody was added and incubated for 3 h at room temperature. Then, 100 µL of substrate (TMB) solution was added and incubated for 15 min protected from light. The reaction was stopped by the addition of 50 µL of stop solution. Optical density (OD) values were measured at 450 nm. The sensitivity of the assay was 1.56 ng/mL.

On day 10, the mice were euthanized, and the ankle joints were removed and fixed for 12 h in 10% paraformaldehyde (pH 7.2), subsequently incubated in 10% EDTA, pH 7.2, for 10 days at room temperature to decalcify the bone, washed with PBS, dehydrated, embedded in paraffin, sliced into 3-µm-thick sections, and stained with hematoxylin and eosin (H&E). To eliminate potential bias, the slides were scored by two independent observers. The sections were subjectively graded using the following parameters: synovial hyperplasia (pannus formation) severity, cellular exudates, cartilage depletion/bone erosion (each scored 0 [normal] to 3 [severe]), and extent of synovial infiltration (scored 0–5, with higher scores indicating greater infiltration). The grades for all parameters were subsequently summed to obtain an arthritis index, with results expressed as the median arthritis score (**Figure 8**).

### CAIA Mouse Model

Eight–twelve-week-old female C57BL/6 mice were obtained from Charles River Laboratories and housed in the Commissariat à l’Energie Atomique Saclay animal facility for experimentation. To induce CAIA (day 0), mice were intravenously injected with an arthritogenic mAb cocktail comprising four antibodies against collagen II (Arthrogen-CIA 5-Clone Cocktail Kit, Amsbio; 200 mg/kg) under anesthesia as described (25). On day 3, the animals were intraperitoneally injected with LPS (2 mg/kg). On day 5, the mice were randomized into groups based on disease severity and intraperitoneally dosed with vehicle (PBS), IVIg (1,000 mg/kg), LFBD192 (100 mg/kg), or Fc-trimer (100 mg/kg) (single administration). Dexamethasone was daily and subcutaneously administered from day 5 at a dose of 1 mg/kg until the end of the study. The mice were monitored for 11 days for clinical signs of arthritis (**Figure 9A**). An analgesic, buprecare (0.1 mg/kg, administered subcutaneously once a day), was used if mice exhibited signs of discomfort, such as difficulty walking or feeding themselves. Clinical parameters were scored as follows: Arthritis severity in each limb (wrist and ankle) was scored blindly and daily on a scale from 0 to 3 (ankle thickness (mm): score 0: 2.5≥X; score 1: 2.6<X ≤ 2.8; score 2: 2.9<X ≤ 3.1; score 3: X>3.1 and wrist thickness (mm): score 0: 1.8≥X; score 1: 1.9<X ≤ 2.1; score 2: 2.2<X ≤ 2.4; score 3: X>2.4). The final scores corresponded to the sum obtained for each animal paw; each mouse could score a maximum of 12 points (3x4). The measurements were performed vertically for the front legs and horizontally for the rear legs using a digital caliper (Fisherbrand).

### Study Approval

All CAIA experiments were associated with Project No. 17_045 submitted to the Ethics Committee in Animal Experimentation (EAEC) No. 44 and approved by “le ministère de l’éducation nationale, de l’enseignement supérieur et de la recherche” under the number APAFIS # 9410-201703271734338. All K/BxN experiments were conducted in the Artimmune Laboratory (Orléans, France) and performed in compliance with the guidelines of the French Ministry of Agriculture for experiments with laboratory animals (law 87-848). All animal experiments were approved by the “Ethics Committee for Animal Experimentation of CNRS Campus Orleans” (CCO) under number CLE CCO 2015-1081.

## Results

### Design and Screening of Fc Variants Optimized for hFcγR and hFcRn Binding

We have previously identified key mutations in the Fc region of human IgG1 that increased hFcRn-binding capacity at pH 6.0 using a fully randomized mutagenesis approach combined with a pH-dependent phage display selection process (26). Similar approaches, but including directed mutagenesis, were used to design human IgG1 Fc variants with increased capacity for binding to hFcγRIIIa (unpublished data), which allowed to select one lead variant—A3A-184A (K334N/P352S/A378V/V397M)—and two key mutations, Y296W and K290G.

To obtain optimized Fc fragment variants with increased binding affinity for both hFcRn and hFcγRIIIa, we attempted two strategies: one involved increasing hFcγRIIIa binding for the previously described hFcRn-optimized variants C6A-74 (V259I/N315D/N434Y) and T5A-74 (N315D/A330V/N361D/A378V/N434Y) (26), the other involved increasing hFcRn-binding at pH 6.0 for the hFcγRIIIa-optimized variant A3A-184A. The screening process was done by Bio-Layer Interferometry (BLI) with the Octet system (PALL) comparing binding ratios of different Fc variants *vs* wild-type Fc related to hFcRn and hFcγRIIIa. Biacore measurements were then performed for the final characterization of the selected lead.

Eight Fc fragment variants were designed, produced in mammalian cells (FreeStyle 293-F cells), and tested for their hFcRn and hFcγRIIIa binding capacity by BLI (at pH 6.0 and 7.4, respectively, Octet system, PALL) together with the non-mutated Fc fragment (wild-type Fc fragment; rFc-WT) and the three lead variants (rFc-C6A-74, rFc-T5A-74, and rFc-A3A-184A). Compared to our previous observations [ (27) and unpublished data], the three lead variants produced as Fc fragments exhibited binding ratios similar to those of other IgG1 formats (**Table 1**). For instance, the rFc-C6A-74 and rFc-T5A-74 variants displayed an optimized ability to bind hFcRn, and not hFcγRIIIa, with increased ratios of 5.6 and 9.0, respectively, compared with that for rFc-WT. As expected, the rFc-A3A-184A variant showed an optimized capacity for binding to hFcγRIIIa, and not hFcRn, with an increased ratio of 3.9.

**Table 1:** KD determined by BLI (Octet device) of 8 new mutants *vs* initial variants at pH6.0 for hFcRn and pH7.4 for hFcγRIIIa (KD determined as means of 3 experiments).

Molecule	Mutations	KD (nM)			Ratio KD WT/variant		
		hFcRn	SD	hFcγRIIIa	SD	hFcRn	hFcγRIIIa
rFc-WT		36.5	8.2	504	75.0	1.0	1.0
rFc-C6A-74	V259I/N315D/N434Y	6.4	0.5	352	44.5	5.6	1.4
rFc-C6A-74W	V259I/Y296W/N315D/N434Y	12.3	0.4	325.5	48.8	2.9	1.5
rFc-C6A-74G	K290G/V259I/N315D/N434Y	9.5	0.9	234	15.6	3.8	2.2
rFc-T5A-74	N315D/A330V/N361D/A378V/N434Y	4	1.9	317	45.3	9.0	1.6
rFc-T5A-74A	N315D/N361D/A378V/N434Y	4.1	1.3	337	42.1	8.8	1.5
rFc-T5A-74MA	Y296W/N315D/N361D/A378V/N434Y	3.6	1.8	217	4.9	10.0	2.3
rFc-T5A-74AG	K290G/N315D/N361D/A378V/N434Y	18.9	1.9	165	15.6	1.9	3.1
rFc-A3A-184A	K334N/P352S/A378V/V397M	29.4	2.2	129	5.7	1.2	3.9
rFc-A3A-184E	Y296W/K334N/P352S/A378V/V397M	53.8	4.9	122	26.9	0.7	4.1
rFc-A3A-184AG	K290G/K334N/P352S/A378V/V397M	23.6	8.5	448	66.5	1.5	1.1
rFc-A3A-184AY	K334N/P352S/A378V/V397M/N434Y	7.8	0.0	132	14.1	4.6	3.8

The T5A-74 variant was first simplified by removing the A330V mutation (T5A-74A variant), which was shown to selectively decrease its binding to C1q but not to other Fc receptors (27) and unpublished data]. Indeed, the rFc-T5A-74 and rFc-T5A-74A variants exhibited similar binding capacities for hFcRn and hFcγRIIIa. The Y296W and K290G mutations, previously selected on hFcγRIIIa, were added to the three mutants: C6A-74 (rFc-C6A-74W and rFc-C6A-74G), T5A-74A (rFc-T5A-74MA and rFc-T5A-74AG), and A3A-184A (rFc-A3A-184E and rFc-A3A-184AG). The Y296W mutation further improved hFcγRIIIa for all variants but at the expense of hFcRn binding for the rFc-C6A-74 and rFc-A3A-184A variants, whereas the addition on the rFc-T5A-74 variant had little effect on hFcRn binding. Similarly, the addition of the K290G mutation had diverse effects depending on the parental variant, i.e., it increased binding to hFcγRIIIa but decreased binding to hFcRn when added to the rFc-C6A-74 and rFc-T5A-74A variants and decreased binding to both Fc receptors when added to the rFc-A3A-184A variant. These results highlighted the difficulty of rational design of the variants, as mutations can have adverse effects depending on the mutations already introduced. Finally, the N434Y mutation, which was used in both the hFcRn-optimized rFc-C6A-74 and rFc-T5A-74 variants and was localized in the hFcRn binding site, was added to the rFc-A3A-184A variant. The resulting combination variant rFc-A3A-184AY exhibited the best binding properties towards both hFcRn and hFcγRIIIa (**Table 1**).

**Figure 1:**
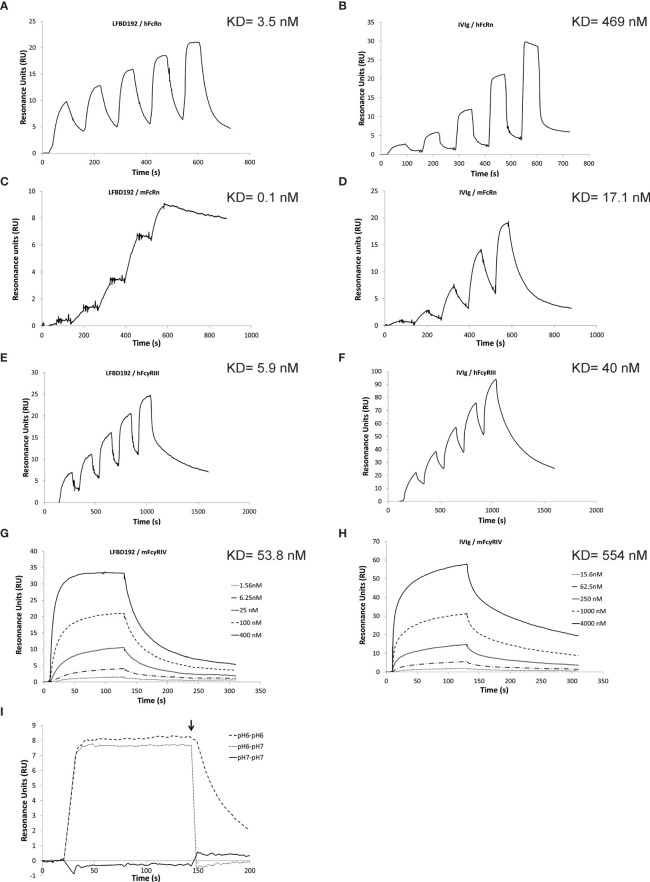
Biacore analysis of LFBD192 binding to hFcRn and hFcγRIIIa. Representative surface plasmon resonance (SPR) sensorgrams obtained for **(A)** the injection of LFBD192 at 4.7–75 nM on ~25 resonance units (RUs) of immobilized hFcRn using phosphate at pH 6.0; **(B)** the injection of IVIg at 24.7–2,000 nM on ~25 RUs of immobilized hFcRn using phosphate at pH 6.0; **(C)** The injection of LFBD192 at 0.2–20 nM on ~50 RUs of immobilized mFcRn using phosphate buffer at pH 6.0; **(D)** The injection of IVIg at 3.7–300 nM on ~50 RUs of immobilized mFcRn using phosphate buffer at pH 6.0. **(E)** The injection of LFBD192 at 6.25–100 nM on ~40 RUs of immobilized hFcγRIIIa using Tris buffer at pH 7.4; **(F)** the injection of IVIg at 31.25–500 nM on ~80 RUs of immobilized hFcγRIIIa using Tris buffer at pH 7.4; **(G)** The injection of LFBD192 at 1.56–400 nM on ~40 RUs of immobilized mFcγRIV using Tris buffer at pH 7.4; **(H)** the injection of IVIg at 15.6–4,000 nM on ~40 RUs of immobilized mFcγRIV using Tris buffer at pH 7.4; **(I)** 100 nM injections of LFBD192 on 14 RUs of immobilized hFcRn in phosphate buffer pH 7.4 followed by a dissociation phase at pH 7.4 (solid line), phosphate buffer pH 6.0 followed by a dissociation phase at pH 6.0 (dotted line), or phosphate buffer pH 6.0 followed by a dissociation phase at pH 7.4 (dashed line) using dual injection mode. The end of the association phase is indicated by an arrow.

Based on these results, the Y296W mutation was added to the rFc-A3A-184AY variant to further improve its binding affinity for hFcγRIIIa, while maintaining its optimization for hFcRn, yielding our lead variant, named LFBD192 (Y296W/K334N/P352S/A378V/V397M/N434Y). Real-time surface plasmon resonance (SPR) measurements were then performed with Biacore to analyze the LFBD192 interaction *vs* IVIg at pH 6.0 with hFcRn and mFcRn and at pH 7.4 with hFcγRIIIa and its mouse orthologue mFcγRIV (**Figure 1** and **Table 2**). A comparative analysis of the sensorgrams showed different behaviors for IVIg and LFBD192 (**Figure 1**). The fast association/dissociation phase observed for the interaction between IVIg and hFcRn agreed best with the steady-state interaction model, while LFBD192 interaction followed a simple first-order (1:1) Langmuir model. As indicated by the *K*_D_ values, the LFBD192 affinity for hFcRn was more than two orders of magnitude higher than that of IVIg (3.5 nM *vs* 469 nM, respectively) (**Figure 1**). Furthermore, SPR experiments were conducted to analyze the pH dependency of the interaction between LFBD192 and hFcRn. As shown in **Figure 1**, LFBD192 is bound to hFcRn at pH 6.0, but not at pH 7.4. Once a complex was formed at pH 6.0, LFBD192 dissociated instantly from hFcRn when the pH was shifted to 7.4, whereas a slower dissociation rate was observed at pH 6.0.

**Table 2:** Equilibrium dissociation constants of LFBD192 *vs* IVIg regarding hFcRn, mFcRn, hFcγRIIIa and mFcγRIV at pH6.0 for FcRn (mouse and human) and pH7.4 for hFcγRIIIa and mFcγRIV, χ2 values are provided as an assessment of the goodness of fit.

Sample	hFcRn			mFcRn			hFcγRIIIa			mFcγRIV		
	KD (nM)	CV (%)	χ^2^	KD (nM)	CV (%)	χ2	KD (nM)	CV (%)	χ2	KD (nM)	CV (%)	χ2
**LFBD192**	3.5^a^	17	0.5	0.1^a^	12.6	0.2	5.9^c^	21	0.4	53.8^a^	8.8	0.6
**IVIg**	469^b^	9.8	0.1	17.1^a^	4.2	7.6	40^a^	0.7	6.9	554^a^	15.2	1.5

a The kinetic constant was obtained using the simple first-order (1:1) Langmuir bimolecular interaction model.

b The affinity constant was obtained using the steady state model.

c The kinetic constant was obtained using the two state reaction model. The use of this model has been demonstrated by a linked reaction test and a linear Scatchard plot (data not shown).

LFBD192 and IVIg also behaved differently in their interaction with mFcRn. Although both interactions followed a first-order (1:1) Langmuir model, LFBD192 had a significantly lower off rate, resulting in a more than two orders of magnitude higher affinity for mFcRn compared with IVIg (**Figures 1C, D**). Our results showed that LFBD192 has a higher affinity than IVIg for both human and mouse FcRn receptors. Also, the affinity of LFBD192 for mFcRn was 35 times higher than that for the human receptor, a difference that was driven by the low dissociation rate.

For the interaction with hFcγRIIIa, IVIg followed a simple first-order (1:1) Langmuir model, while a biphasic dissociation phase was observed for LFBD192, which rendered the use of the (1:1) Langmuir model unsuitable for the analysis (**Figures 1E, F**). The choice of the two-state reaction model was supported by the linked test results, which showed a linked reaction (the dissociation rate is dependent on the contact time), and a Scatchard plot, which showed a linear behavior favoring the existence of a single binding site on the analyte. This observation indicated that a conformational change might stabilize the LFBD192-hFcγRIIIa complex during the interaction, resulting in nearly one log_10_ higher affinity compared with IVIg.

The interaction of LFBD192 and IVIg with mFcγRIV both followed a simple first-order (1:1) Langmuir model (**Figures 1G, H**). The affinity of LFBD192 for mFcγRIV was nine-fold lower than that for hFcγRIIIa; however, the increase in the affinity of LFBD192 in comparison with IVIg was proportionally similar for mFcγRIV and hFcγRIIIa.

The LFBD192 produced by CHO-S (Freedom CHO-S) and purified in a 2-step chromatography process was monomeric with an apparent molecular weight of 51.4 kDa, as determined by size-exclusion chromatography. LC-MS analysis of the deglycosylated and reduced LFBD192 resulted in two main forms, namely, an intact Fc (25658 Da) and a C-terminal lysine-clipped form (25531 Da), which validated the polypeptide sequence (theoretical 25656 Da). N-glycan analysis revealed biantennary structures composed primarily of G0F glycan species, which are commonly found in CHO-derived antibodies (data not shown).

### LFBD192 Blocked Anti-Fc Receptor Molecules on the Different hFcγRs with High Efficacy

To further explore the interaction between LFBD192 and the Fc receptors, the ability of both LFBD192 and IVIg to compete with a mAb specific for hFcγRIIIa (3G8 mAb) or a generic mAb competing for Fc receptors through its Fc portion were compared in a competitive binding assay using the different human Fc gamma receptors expressed on the surface of cells from different lines, namely, Jurkat-FcγRIIIa-F158, HEK-FcγRIIa-H131, HEK-FcγRIIa-R131, HEK-FcγRIIb, and Jurkat-FcγRI. LFBD192 has been compared to several molecules: IVIg widely used to treat immune complex driven inflammatory diseases, a modified Fc (Fc-MST-HN) targeting only FcRn (2) and a trivalent Fc (Fc-trimer) described to bind with high avidity to all Fc receptors (30). The two latter molecules were *in-house* designed according to information provided in publications (2, 30) and patents (WO 2016142782 and WO 2015168643) and produced in mammalian cells (HEK293). The competition assay was performed between LFBD192, IVIg, Fc-MST-HN, and the Fc-trimer and two different labeled IgG1 as anti-Fc receptors depending on the Fc receptors studied (**Table 3**). Rituximab was used for HEK-FcγRIIa-H131, HEK-FcγRIIa-R131, HEK-FcγRIIb, and Jurkat-FcγRI and the 3G8 anti-FcγRIIIa mAb was used for Jurkat-FcγRIIIa-F158. Experiments with FcγR-transfected cells were performed at pH 7.4. As expected, LFBD192 was more potent than IVIg in its ability to inhibit a competitor IgG1 (**Table 3**). The IC50 of IVIg against FcγRIIIa-F158, FcγRIIa-R131, FcγRIIb, and FcγRI was 10-15-fold higher than that of LFBD192. The IC50 of LFBD192 *vs* IVIg was lower (4.5 times) for the FcγRIIa variant H131. Fc-MST-HN exhibited no inhibitory activity toward the FcγRs, except for FcγRI. In contrast, the trimeric Fc avidity allowed a higher potency compared to LFBD192 one for FcγRs except for FcγRI.

**Table 3:** Determination of inhibitory concentration at 50% (IC 50) between IgG1 and LFBD192, IVIg, Fc-MST-HN, and Fc-trimer on cell lines transfected with hFcRn or hFcRs (IC50 calculated with mean of 2 experiments).

Transfected cell line	IgG	LFBD192	IC 50 (nM)		
			IVIg	Fc-MST-HN	Fc-trimer
Jurkat-FcRn	Rituximab	15	1356	14	23
Jurkat-FcγRIIIa-F	3G8 mab	123	1684	>2170	39
HEK-FcγRIIa-H	Rituximab	147	671	>2170	12
HEK-FcγRIIa-R	Rituximab	132	1308	>2170	17
HEK-FcγRIIb	Rituximab	55	761	>2170	16
Jurkat-FcγRI	Rituximab	70	880	494	208

### LFBD192 Blocked Anti-Fc Receptor Molecules on FcRn with High Efficacy

**Figure 2:**
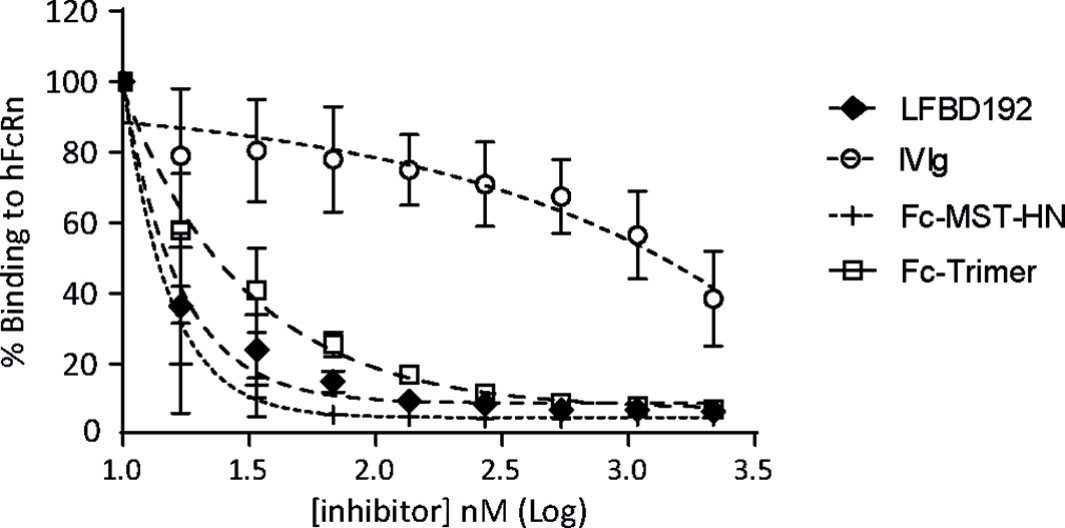
LFBD192 blocks the binding of IgG to FcRn. The binding of LFBD192, IVIg, Fc-MST-HN, and the Fc-trimer to human FcRn expressed at the surface of Jurkat cells measured at pH 6.0 in a competitive assay in the presence of Alexa-labeled rituximab (30 µg/mL) (data represent the means ± SEM of two experiments, each experiment performed in triplicates).

LFBD192 interactions with FcRn receptors and competition with immunoglobulins were explored using a Jurkat cell line that overexpressed FcRn (**Figure 2**). The ability of LFBD192 to compete with an antibody (rituximab) was compared to three molecules at pH 6.0: IVIg, Fc-MST-HN that targets only FcRn (2), and Fc-trimer, reported to bind with high avidity to all Fc receptors (25). The strongest inhibitory effect was observed for LFBD192 and Fc-MST-HN (**Figure 2**). The LFBD192 and Fc-MST-HN IC50 ratios *versus* IVIg were 90 and 96 times, respectively (**Table 3**). Surprisingly, the Fc-trimers with an IC50 value of 23 were not as efficient as LFBD192 (IC50 = 15) or Fc-MST-HN (IC50 = 14).

### LFBD192 Protects Against IC-Mediated Cell Activation

**Figure 3:**
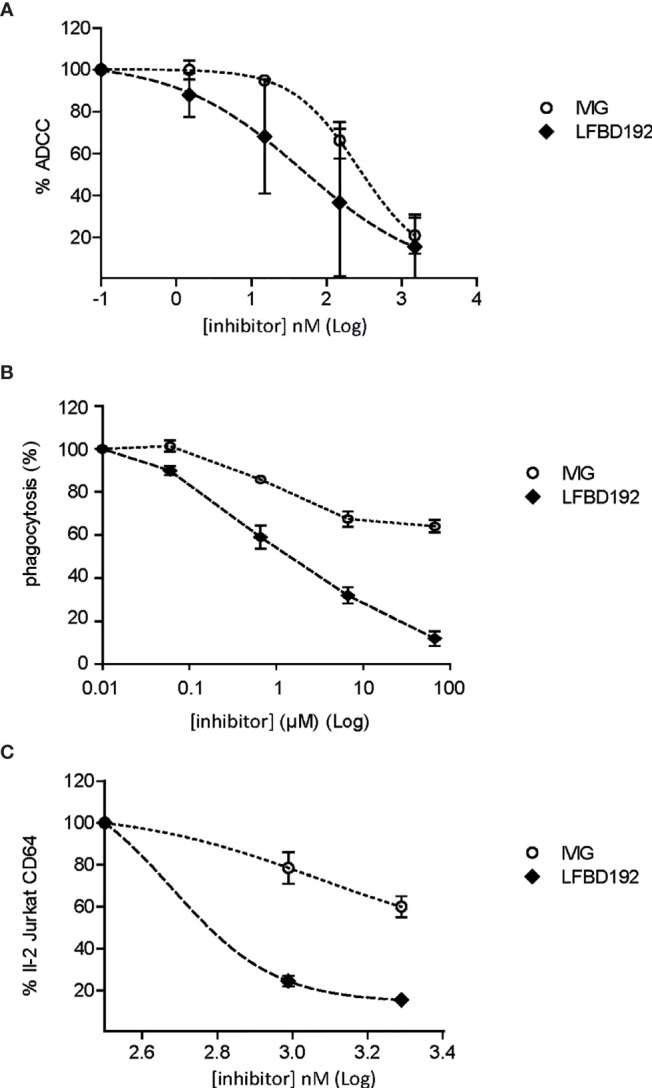
LFBD192 *vs* IVIg in three models of inhibition of immune complex-mediated cell lysis. **(A)** Percentage of antibody-dependent cell-mediated cytotoxicity (ADCC) inhibition for anti-D antibody-treated O^+^ human red blood cells (data are the means of two experiments from different human donors, each experiment performed in triplicates). **(B)** Percentage of phagocytosis inhibition of human anti-platelet IgG1 (anti-CD41)-treated fluorescence-labeled platelets obtained from human donors mediated by FcγRIIIa-transfected THP1 cells with 6.66×10^–5^, 6.66×10^–6^, 6.66×10^–7^, and 6.66×10^–8^ M of 10% IVIg or LFBD192 (data represent the means ± SEM of three experiments from three different human donors, each experiment performed in duplicates). **(C)** Percentage of inhibition of FcγRI-mediated IL-2 secretion by FcγRI-expressing Jurkat T cell lymphomas (CD20-negative) in co-culture with CD20-expressing Raji cells rituximab/Raji cells complex (data represent the means ± SEM of two experiments from different human donors, each experiment performed in triplicates).

The ability of LFBD192 to protect against antibody-targeted cell lysis by effector cells or effector cell activation was tested in three different *in vitro* models. In the first two models, the ability of LFBD192 to protect against anti-D-mediated red blood cell lysis (**Figure 3A**) and antibody-mediated platelet phagocytosis was tested (**Figure 3B**). Red blood cells were incubated with an anti-D mAb to obtain anti-D-opsonized red blood cells and the ability of the tested molecules to inhibit human PBMC effector-mediated lysis was determined. LFBD192 showed the greatest protective effect with an IC50 of 97 *vs* 351 for IVIg. In the second test, THP1 CD16^+^ cells were incubated with human monoclonal anti-platelet IgG1 (anti-CD41) and fluorescently labeled platelets obtained from human donors (**Figure 3B**). Interestingly, LFBD192 at 0.6 µM demonstrated the same inhibitory effect as IVIg at 60 µM, suggesting that LFBD192 was 100 times more potent than IVIg at inhibiting human antibody-mediated platelet phagocytosis (**Figure 3B**). The observed inhibitory effects were mediated by FcγR blocking.

The ability of LFBD192 to inhibit IC-mediated cell activation was also tested. CD20-expressing Raji target cells were incubated with rituximab in the presence of Jurkat cells overexpressing FcγRI. After 16 h, the amount of IL-2 released by the Jurkat cells was measured. LFBD192 was found to be substantially more potent than IVIg at blocking IC engagement (**Figure 3C**). Indeed, the LFBD192 inhibitory concentration at 25% was 2.5-fold higher than that of IVIg (442 *vs* 1106 nM), and the IC50 of LFBD192 was 600 nM; however, the IVIg IC50 could not be calculated (over 1950 nM).

### LFBD192 Does Not Trigger Immune Reactions *In Vitro*

**Figure 4:**
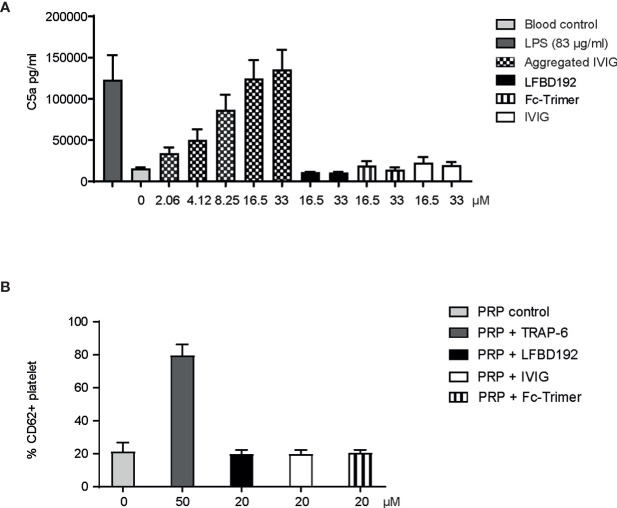
LFBD192 does not trigger *in vitro* immune reactions relating to complement and platelet activation. **(A)** C5a generation in human whole blood in response to lipopolysaccharide (LPS) (83 µg/mL), aggregated IVIg (2.06, 4.12, 6.25, 16.5, 33 µM), LFBD192 (16.5 and 33 µM), Fc-trimer (16.5 and 33 µM) and IVIg (16.5 and 33 µM) was quantified by ELISA. Data are mean ± SEM of 4 experiments from 4 different human donors, each experiment performed in duplicates. **(B)** Platelet activation in response to TRAP-6 (50 µM, positive control), LFBD192 (20 µM), and IVIg (20 µM). Bars represent the means of CD62P expression ( ± SD) as measured by flow cytometry of 2 experiments from 2 different human donors, each experiment performed in duplicates.

**Figure 5:**
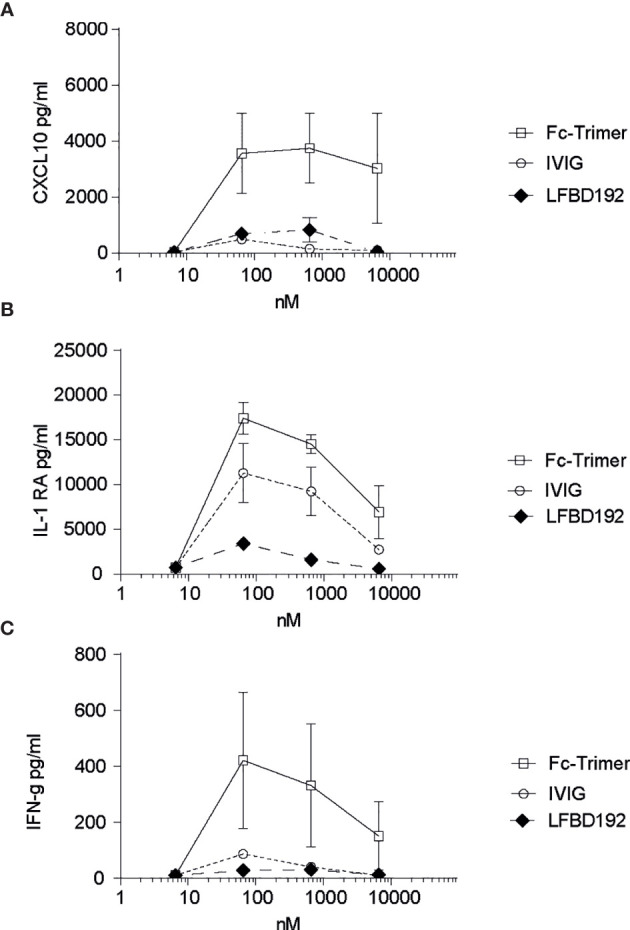
LFBD192 does not trigger cytokine release. Cytokine release in human whole blood was determined by multiplex immunoassay after incubation with LFBD192, the Fc-trimer, and IVIg at 6.5, 65, 650, and 6,500 nM. The pro-inflammatory cytokines G-CSF, IL-1β, IL-12/p70, TNF-α, CXCL9/MIG, and IL-6 and the anti-inflammatory cytokines IL-4, IL-5, IL-10, and IL-22 were not detected. The detected cytokines were CXCL10 **(A)**, IL-1RA **(B)**, and IFNγ **(C)**. Data represent the means ± SEM of two experiments from two human donors, each experiment performed in duplicates.

We next addressed a safety consideration regarding potential complement and platelet activation and FcγR cross-linking that could trigger immune cell activation and cytokine release similar to that observed with IgG aggregates (**Figures 4**, **5**). The potential activation of the complement cascade was assessed by the determination of C5a concentration (**Figure 4A**). LFBD192 (16.5 and 33 µM) was incubated with human whole blood and its ability to induce C5a generation was compared to that of LPS), Fc-trimer (16.5 and 33 µM) aggregated IVIg (2.06, 4.12, 6.25, 16.5, 33 µM)), and IVIg (22.7 µM). Unlike LPS and aggregated IVIg, which served as positive controls, LFBD192 did not generate an increase of C5a in whole blood.

Platelets expressing FcγRIIa could potentially be activated by engagement with LFBD192 optimized for binding to this receptor. Potential platelet activation was tested by incubating LFBD192 or IVIg in PRP from 2 healthy blood donors and then determining the expression levels of CD62P, a platelet activation marker (p-selectin) (**Figure 4B**). Neither LFBD192 nor IVIg at 20 µM induced the expression of CD62P, in contrast to that observed with incubation with the positive control TRAP-6. These results demonstrated that, although LFBD192 was optimized for FcγRIIa binding, it did not promote platelet activation.

To investigate if LFB192 could induce a cytokine release syndrome, increasing concentrations of LFB192 (6.5, 65, 650, and 6,500 nM) were incubated with human whole blood, and the effects on the concentrations of a variety of cytokines were compared with those of incubation with the same concentrations of IVIg and Fc-trimer (**Figure 5**). Importantly, no expression of most of the cytokines assessed (pro-inflammatory G-CSF, IL-1β, IL-12/p70, TNF-α, CXCL9/MIG, and IL-6 and anti-inflammatory IL-4, IL-5, IL-10, and IL-22) was detected after 24 h of incubation (data not shown). Only low levels of the pro-inflammatory cytokines CXCL10 and IFN-γ and the anti-inflammatory cytokine IL-1RA were observed, but at a similar or lower level than with IVIg or the Fc-trimer.

In summary, LFBD192 showed a good *in vitro* safety profile relating to platelet and complement activation. Furthermore, in an *in vitro* human whole blood safety assay, incubation with LFBD192 did not result in cytokine release.

### LFBD192 Potency in Models of Autoimmune Disease

**Figure 6:**
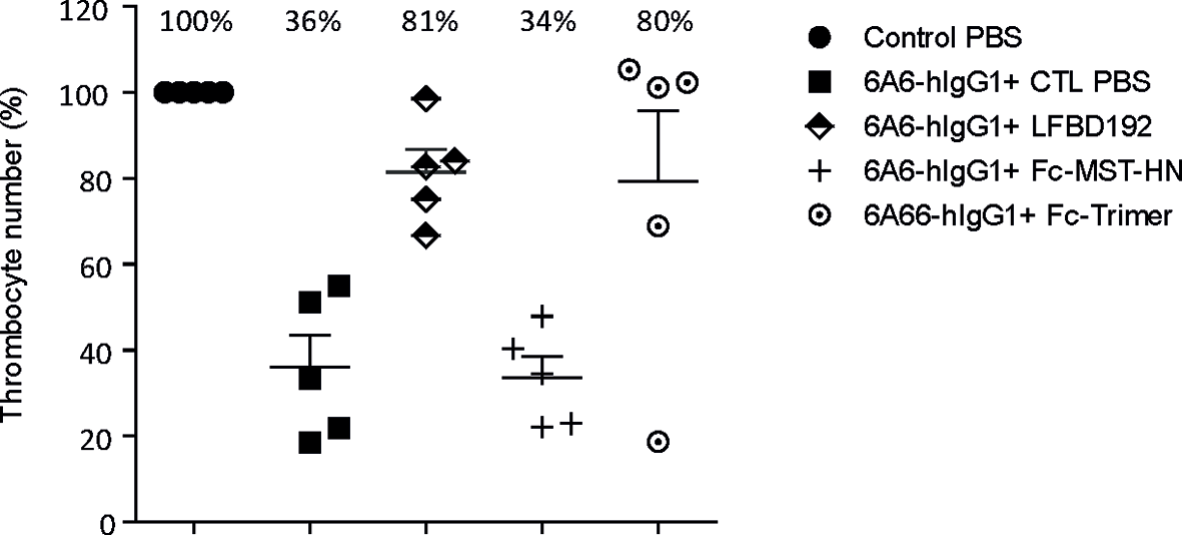
LFBD192 displayed high potency in the prophylactic treatment of ITP in a humanized FcRn mouse model. Platelet numbers 24 h after intraperitoneal injection of anti-platelet 6A6-hIgG1 antibody and treatment with PBS, LFBD192, FC-MST-HN, and the Fc-trimer at 50 mg/kg 2 h before ITP induction. Data represent mean thrombocyte percentages ± SEM (*N*=5).

**Figure 7:**
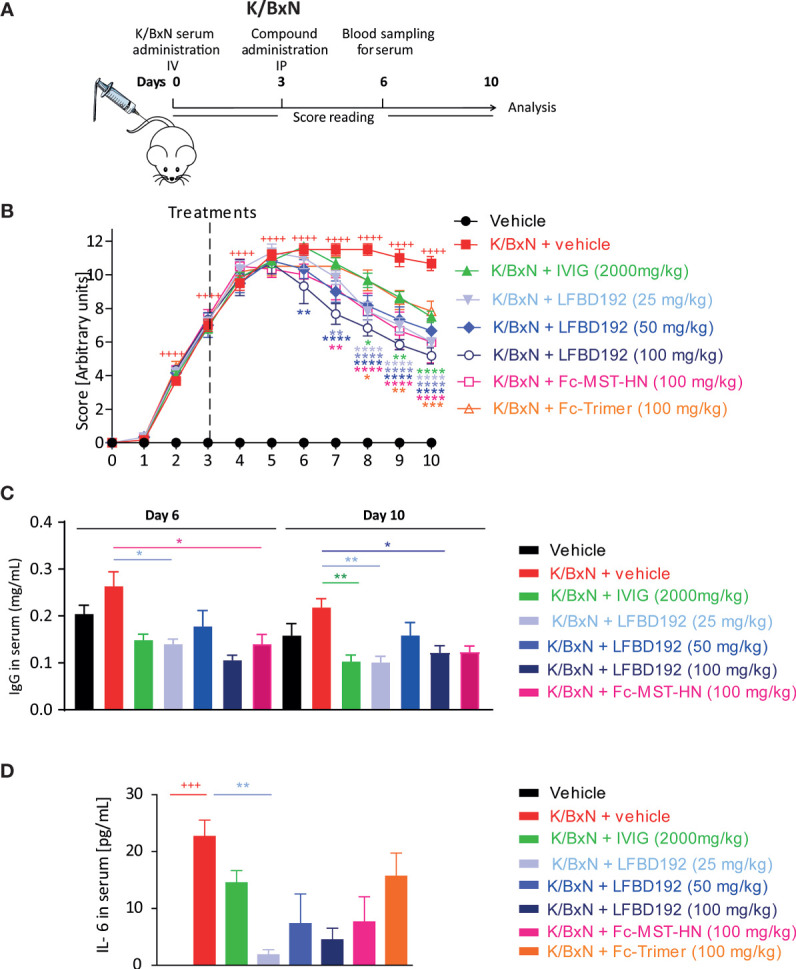
LFBD192 ameliorated disease activity in a K/BxN-induced mouse model of acute arthritis. **(A)** Schematic representation of the mouse K/BxN model with administration of the compound 3 days after K/BxN serum injection. **(B)** Clinical scores after treatment with PBS, IVIg (2,000 mg/kg), LFBD192 (25, 50, and 100 mg/kg), Fc-MST-HN (100 mg/kg), and the Fc-trimer (100 mg/kg). **(C)** Serum IgG levels were reduced at days 6 and 10 in LFBD192-treated mice compared with mice treated with PBS. Bars represent mean serum IgG levels ± SEM (*N*=6 mice per group). This result is representative of at least two experiments. **(D)** The levels of systemic IL-6 were reduced at day 6 in LFBD192-treated mice. Bars represent mean serum IL-6 levels ± SEM (*N*=6 mice per group). This result is representative of at least two experiments. Statistical evaluation of the differences among experimental groups was performed by two-way ANOVA followed by Dunnett’s post-test. *P*-values <0.05 were considered significant. ^+++^ *p*<0.001, ^++++^ *p*<0.0001: vehicle *vs* K/BxN + vehicle; *p<0.05, **p<0.01, ***p<0.001, ****p<0.0001: compared with K/BxN + vehicle. All statistical analyses were performed using GraphPad Prism, version 8 for Windows (GraphPad Software Inc., San Diego, CA, USA).

**Figure 8:**
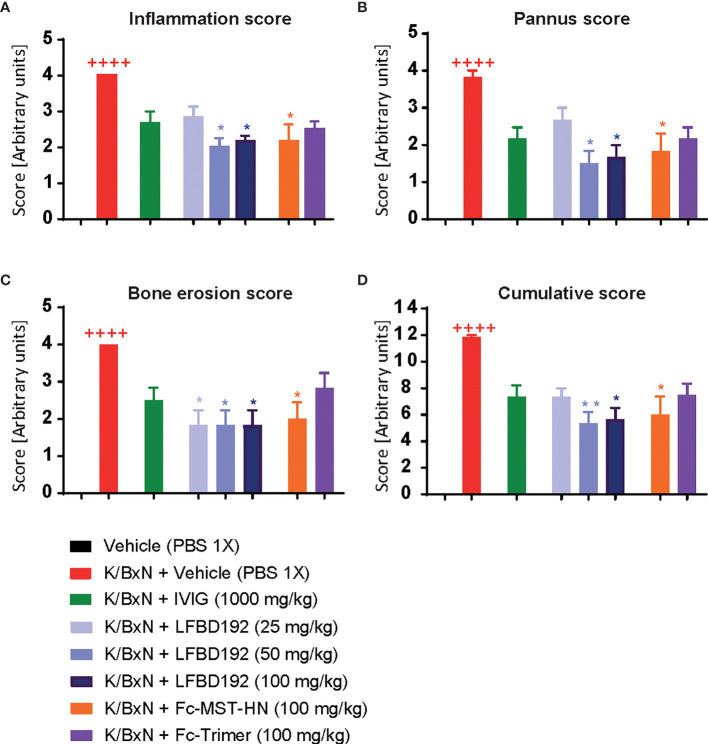
LFBD192 ameliorates histological scores in a K/BxN mouse model. Effect of compounds IVIg (2000mg/kg), LFBD192 (25, 50 and 100mg/kg), Fc-MST-HN (100mg/kg) and Fc-trimer (100mg/kg) on histological scores. On day 10, mice were killed, and ankle joints were harvested and fixed in 10% paraformaldehyde and decalcified for 10 days. Paraffin embedded sections were stained with hematoxylin & eosin (H&E) and scanned under a microscope. Histologic scoring including inflammation **(A)**, bone erosion **(B)**, pannus **(C)**, and cumulative scores **(D)** are shown. The sections were graded using various parameters, as follows: severity of synovial hyperplasia (pannus formation), cellular exudates (inflammation), and cartilage depletion/bone erosion, each scored 0 [normal] to 5 [severe], and extent of synovial infiltrate, scored 0–5, with higher scores indicating greater infiltration. The grades for all parameters were subsequently summed to obtain the cumulative score. Quantitative data show mean and SEM from six animals per group from three independent experiments. Statistical analysis: Non parametric Kruskal-Wallis test followed by a Dunn**’**s multiple comparisons test ^++++^ *p*<0.0001, Vehicle compared with K/BxN + Vehicle; *p<0.05, **p<0.01, K/BxN + Vehicle compared with treatments.

LFBD192 efficacy was tested *in vivo* in three different antibody-mediated autoimmune mouse models, one as preventive treatment (ITP model; **Figure 6**) and two as therapeutic administration (K/BxN and CAIA; **Figures 7**, **8**, respectively). In all tests, LFBD192 was compared to IVIg, Fc-MST-HN, and the Fc-trimer (30). The optimized affinity of LFBD192 for mouse Fc receptors (mFcRn and mFcγRIV) allowed validating of the testing in a mouse Fc receptor context. An ITP model was induced in humanized FcRn mice by intraperitoneal injection of a mouse anti-platelet antibody containing a human Fc fragment (6A6-hIgG1) (15) as a platelet depleting antibody (**Figure 6**). As expected, the depleting antibody (0.3 µg/g) in the presence of PBS (which served as a negative control) elicited a 36% decrease in thrombocyte number. Interestingly, LFBD192 administration at 50 mg/kg 2 h before platelet depletion restored the platelet count to 81%, similar to that seen with the Fc-trimer (80%). In contrast, at the same dose, Fc-MST-HN could not restore platelet levels, suggesting that targeting only FcRn was not sufficient to protect platelets in this ITP prevention model. The 4 and 24 h study points were probably too short to allow the observation of the physiological effect of lowering anti-platelet antibody levels *via* FcRn blocking.

The effect of LFBD192 was also tested in two mouse models of acute inflammatory arthritis, in which ICs and FcγRs have been shown to be implicated (1, 14, 15). The therapeutic efficacy of LFBD192 was first evaluated in the K/BxN-induced arthritis model (**Figure 7**). Inflammation was induced in mouse joints *via* the transfer of serum from arthritogenic mice to C57-BL6 mice. Three days after serum injection, the mice were treated with LFBD192, IVIg, Fc-MST-HN, or Fc-timer (**Figure 7A**). All the tested compounds significantly reduced clinical scores compared with those obtained for the K/BxN + vehicle group (**Figure 8B**). A dose-dependent reduction in clinical scores was observed in mice treated with LFBD192 at 25, 50, and 100 mg/kg (**Figure 7B**). A slightly higher reduction in clinical scores was observed for LFBD192 when compared with the Fc-trimer and Fc-MST-HN at 100 mg/kg and IVIg at 2,000 mg/kg; however, the reductions were not statistically significant. At 25 mg/kg, LFBD192 elicited a reduction in clinical scores equivalent to that for Fc-MST-HN at 100 mg/kg.

Total circulating mouse IgG dosages were determined at days 6 and 10, three and seven days after drug treatments, respectively. The levels of endogenous IgGs were higher in non-treated mice with K/BxN-induced arthritis than in healthy control. LFBD192 at 25 and 100 mg/kg doses, IVIg at 2,000 mg/kg, and Fc-MST-HN at 100 mg/kg elicited a more than 50% reduction in circulating mouse IgG relative to non-treated K/BxN mice (**Figure 7C**).

The level of the inflammatory cytokine IL-6 was analyzed at the peak of the disease (day 6). LFBD192 at 25 mg/kg promoted a significant reduction in the increase of IL-6 secretion compared with that in the K/BxN + vehicle group (**Figure 7D**). A non-significant reduction in IL-6 concentrations was observed for LFBD192 at 50 and 100 mg/kg, Fc-MST-HN at 100 mg/kg, IVIg at 2,000 mg/kg, and the Fc-trimer at 100 mg/kg.

Histopathological analysis showed that compared with the K/BxN + vehicle group, treatment with LFBD192 (50 and 100 mg/kg) or Fc-MST-HN (100 mg/kg) exerted significant protective effects against inflammation in the joints, synovial tissue expansion, and bone erosion (**Figure 8**). In contrast, IVIg at 2,000 mg/kg and the Fc-trimer at 100 mg/kg did not significantly affect the histological score.

**Figure 9:**
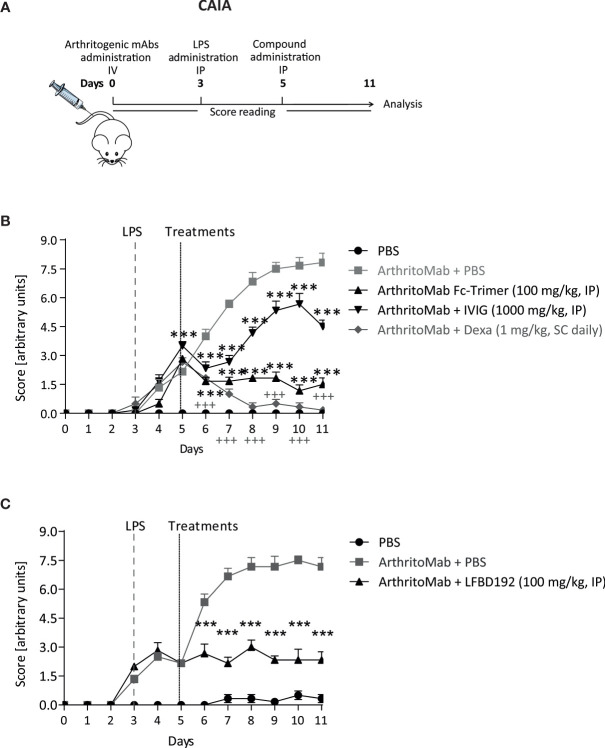
LFBD192 displayed high potency in a mouse model of collagen antibody-induced arthritis (CAIA). **(A)** Schematic representation of the mouse model of CAIA with administration of the compound 5 days after anti-collagen antibody injection. **(B)** Clinical scores after treatment with PBS, the Fc-trimer (100 mg/kg), IVIg (1,000 mg/kg), and dexamethasone (1 mg/kg, daily). **(C)** Clinical scores after treatment with PBS and LFBD192 at 100 mg/kg. Data represent the means ± SEM of 6 mice per group. Statistical evaluation of the differences among the experimental groups was performed using two-way ANOVA followed by Bonferroni’s post-test. *P*-values <0.05 were considered significant. ^+++^ *p*<0.001, Vehicle compared with CAIA + Vehicle; *p<0.05, **p<0.01, ***p<0.001, ****p<0.0001: compared with CAIA + vehicle. All the statistical analyses were performed using GraphPad Prism version 8 for Windows (GraphPad Software Inc.).

LFBD192 was also tested in a CAIA model in which inflammation was triggered by the administration of arthritogenic anti-collagen II antibodies (**Figure 9A**). In a first experiment, mice were treated with IVIg at 1000 mg/kg and Fc-trimer at 100 mg/kg 5 days after antibody injection and daily with 1 mg/kg dexamethasone (**Figure 9B**). The dose for each molecule was determined to be comparable to that previously reported (25). The mice from the group injected with arthritogenic antibodies without treatment developed acute arthritis, reaching a clinical score close to 7.5. From day 6, all treatments decreased the clinical scores, although the Fc-trimer elicited the best results, as previously described (25). In a second experiment, LFBD192 was injected at 100 mg/kg on day 5 led to a marked decrease in the clinical score at day 6 (**Figure 9C**).

Taken together, the results obtained with the three mouse models of autoimmune disease suggested that LFBD192 has 20 to 80-fold greater potency than IVIg. Furthermore, LFBD192 efficacy was slightly higher than that of Fc-MST-HN, an Fc with mutations that allow only FcRn optimization in a preventive ITP model, as well as that of a trivalent Fc in a mouse model of K/BxN-induced arthritis. In the CAIA mouse model, LFBD192 had similar efficacy to the Fc-trimer.

## Discussion

Two mechanisms of action associated with pathogenic autoantibody-antigen ICs can be targeted. They circulate antibody levels and block the inflammatory pathways through a mechanism that seems to involve FcγRs expressed on the surface of effector cells.

As the half-life of IgGs is dependent on FcRn, several molecules (mAbs and FcRn ligands) that target FcRn are currently under development, aiming to reduce the levels of circulating antibodies, including autoantibodies (3, 5). IVIg infusion has been shown to reduce antibody levels by 40% and may aid in reducing inflammation in chronic autoimmune conditions (31, 32). However, the mechanism of action of IVIg is not limited to reducing plasmatic antibody levels. The F(ab′)2 portion of IVIg has been suggested to be involved in its immunomodulatory function as it is responsible for the neutralization of cytokines and autoantibodies, the blockade of cell-cell interactions, and C3a and C5a scavenging (15, 22). Many studies in mice have shown that the Fc portion of IVIg (obtained by digestion) often represents the active component of IVIg *via* interaction with Fc receptors, such as in ITP and arthritis (K/BxN arthritis models) (33–35). The main direct mechanisms of action of the Fc portion of IVIg include FcRn saturation, activating FcγR blockade, and the modulation of activating *vs* inhibitory FcγR expression (15, 22).

The dual targeting of both FcRn and FcγRs seems to be the most promising therapeutic approach. Although clinical data on Fc fragments are encouraging (10, 11), the development of Fc fragments purified from IVIg does not resolve the IVIg supply problem and is too costly for therapeutic use, and a recombinant Fc could be the solution. To greatly reduce the administered dose, several derived Fc fragments have been developed (9). Notably, multivalent Fc molecules bind avidly to all Fc receptors and confer efficacy at much lower doses than IVIg. Highly ordered Fc multimers (stradomer/GL-2045) were shown to have high efficacy in several animal autoimmune models of ITP, collagen-induced arthritis (CIA) (23), myasthenia gravis (36), and experimental autoimmune neuritis (37). Smaller hexameric Fc constructs are also potent in CAIA, CIA, and ITP mouse models (24, 38). Nevertheless, the potential for FcγR cross-linking by multimeric Fc that could trigger immune cell activation and cytokine release similar to that seen with IgG aggregates is a safety consideration that should be addressed.

A multivalent Fc (GL-2045) did not induce statistically significant increases in the levels of any of the well-recognized pro-inflammatory cytokines in NHPs (39). Additionally, the injection of a hexameric Fc molecule in naïve animals elicited only limited cytokine release in mice and none in monkeys (24); however, in human blood safety assays, incubation with IgG1 isotype Fc hexamers resulted in cytokine release. Platelet and complement activation was avoided using Fc from IgG4 (40). Furthermore, trimeric Fc was determined to be the minimal structure to not activate immune effector cells (25). Specifically, pentameric Fc is a strong agonist of FcγR and mediating in SYK and ERK phosphorylation.

Multivalent Fc (GL-2045) and trimeric Fc (CSL-730) safety evaluations are in progress in phase I clinical trials. However, to avoid potential FcγR cross-linking by multimeric Fc, we chose to develop a monovalent Fc, LFBD192, that was modified to have a binding profile similar to that of multivalent Fc.

We have shown that LFBD192 has a good *in vitro* safety profile regarding platelet and complement activation. Furthermore, in a human whole blood safety assay, incubation with LFBD192 did not result in cytokine release. This result contrasts with an IgG1 isotype hexavalent Fc safety concern in a similar test (40). The critical disparity between IgG1 and IgG4 isotype Fc hexamers related to cytokine release was suggested to be linked to their differential ability to bind FcγRs on neutrophils in whole blood, and particularly FcγRIIIb (40). We did not determine binding to FcγRIIIb; however, LFBD192 is bound to neutrophils with slightly lower affinity than that seen with the trivalent Fc. The *in vitro* safety profile of LFBD192 will need to be confirmed in a toxicology study in NHPs.

For the first time, a molecule was engineered that was significantly optimized for binding to the main FcγRs as well as FcRn. A recent review (41) described several mAbs with point mutations that enhance FcγR binding to optimize the effector function of therapeutic mAbs or enhance FcRn binding to optimize the half-life of mAbs. Many examples have been reported where introducing a mutation in the Fc region can optimize the binding to one FcγR while also negatively influencing another (41). None of them has allowed the dual optimization of FcγR and FcRn binding as seen with LFBD192. Furthermore, our mutations have not been described elsewhere. The combination of the six mutations was unpredictable by rational design. The key mutations allowing optimization for FcRn binding were first identified through two successive rounds of random mutagenesis and phage display selection. This process allowed the exploration of a huge number of mutants (1×10^7^ and 2×10^7^, respectively) (26). Furthermore, two rounds of random mutagenesis/phage display selection relating to FcγRIIIa have allowed the identification of new key mutations that modulate the binding to FcγRIIIa (unpublished data). Data obtained from random mutagenesis were used to build a database of molecules designed by rational mutagenesis and binding properties related to the different FcγRs and FcRn (27). Here, we designed new mutants by combining mutations previously identified to optimize binding to FcRn with mutations that optimize binding to FcγRIIIa. Three steps of design/characterization led to the selection of the Y296W/K334N/P352S/A378V/V397M/N434Y mutations in LFBD192. This molecule has a higher binding affinity than IVIg for the main human FcγRs and FcRn as well as for mouse FcRn and FcγRIV. Efgartigimod has recently shown efficacy in reducing serum IgG levels in humans (3) and elicited long-lasting disease improvement in myasthenia gravis patients (4). The mutations in efgartigimod (M252Y/S254T/T256E/H433K/N434F) allowed the optimization of binding only to FcRn at both acidic (*K*_D_: 14.2 nM) and neutral pH (*K*_D_: 320 nM) (3). In addition to being optimized for FcγR binding, LFBD192 also has a strong affinity for FcRn at pH 6.0, similar to efgartigimod. In contrast to efgartigimod, however, LFBD192 has no detectable binding at pH 7.4, allowing the conservation of the pH dependence. In a human clinical trial, efgartigimod has shown efficacy at the dose of 10 mg/kg administered four times over 3 weeks (4). Here, we compared LFBD192 to Fc-MST-HN, an Fc fragment produced *in-house* that contained the same mutations as efgartigimod. LFBD192 and Fc-MST-HN showed similar binding potential for FcRn (IC50 of 15 and 14 nM, respectively); however, Fc-MST-HN displayed a low affinity for FcγRIIa, FcγRIIIa, and FcγRI (data not shown). Fc-MST-HN was not effective in the preventive ITP model at 50 mg/kg, likely because determining platelet counts up to 24h is not long enough to allow the anti-FcRn drug to substantially affect IgG elimination. Notably, in the ITP model, FcRn involvement was studied in the context of human FcRn (humanized FcRn mice and chimeric IgG1 anti-platelet antibody) and mouse FcγRs. FcγRIV being the receptor mainly involved in the ITP mouse model induced through a human IgG1 Fc (6A6-hIgG1), the LFBD192 efficacy *vs* Fc-MST-HN could be explained with a higher binding and then inhibitory of FcγRIV dependent cytotoxicity mechanisms. In the arthritis model, the therapeutic administration of 100 mg/kg of Fc-MST-HN had similar potency to 25 mg/kg LFBD192. In addition, similar to that seen with Fc-MST-HN, LFBD192 could significantly reduce the levels of endogenous IgGs for at least 7 days after drug injection. An MST-HN variant in the IgG format was reported to elicit a similar reduction in circulating IgG in arthritic mice (3). In humans, we could expect to have a similar effect on IgG depletion at the efgartigimod doses with an additional effect on the acute inflammation targeting FcγRs. Although the efficacies of the molecules were assessed in the context of mouse Fc receptors, the increased binding affinity of LFBD192 compared with IVIg for mouse FcRn was shown to be proportionally similar for human FcRn. The higher inflammatory reduction of LFBD192 compared to Fc-MST-HN seems to be due to an inhibitory effect of pathogenic IgG2a through a higher affinity for FcγRIV.

An alternative anti-inflammatory mechanism was described using sialylated antibodies (29, 42, 43). LFBD192 produced in CHO cells is not sialylated, suggesting a different mechanism of action mainly focused on the blockade of type I FcγRs and FcRn.

LFBD192 was also compared with a trivalent molecule (Fc-trimer) designed and produced *in-house* based on the Fc3Y molecule described by Ortiz et al. (25). Fc3Y is currently undergoing a phase I clinical trial under the name CSL730. LFBD192 and the Fc-trimer were shown to have similar efficacy in the preventive ITP model. Fc3Y was reported to be very efficient in the CAIA model (25) and although LFBD192 and Fc-trimer were not compared simultaneously in the same experiment, we obtained comparable efficacy with the Fc-trimer at 100 mg/kg in our CAIA experiment. The slightly lower potency of the Fc-trimer at 100 mg/kg in the K/BxN-induced mouse model of acute arthritis was surprising. The Fc-trimer and IVIg at 2,000 mg/kg showed similar clinical score evolutions and reductions in IL-6 levels. One explanation could be that a correlation exists between the size (50 kDa for monovalent Fc-fragments [LFBD192 and Fc-MST-HN] and 150 kDa for IVIg and the Fc-trimer) and potency of the molecules that could allow better infiltration of the smaller molecules into the inflamed joints.

The risk of immunogenicity due to mutations introduced in a highly conserved human IgG1 Fc sequence could not be excluded. In humans, different modified Fc on mAbs, and, to our knowledge, Fc fusion proteins, have been administered without any reported immunogenicity. The injection of efgartigimod in human volunteers did not trigger the development of anti-drug Ab signals (3). The *in-silico* analysis undertaken by Lonza predicted that our 6 mutations presented only a low risk of immunogenicity. The DRB1 scores for LFBD192 were at the lower end of human marketed antibodies (data not shown).

The conservation of the pH dependence of LFBD192 allowed for the hypothesis that the mechanism of action underlying the effects of LFBD192 on FcγRs involves two steps: LFBD192 first blocks FcγRs on the surface of effector cells at physiological pH 7.4, providing a rapid modulation of inflammation in the acute phase of the disease without being depleted *via* the binding to FcRn expressed on the surface of immune effector cells (monocytes, neutrophils, dendritic cells) and the endothelium. In a second step, LFBD192 saturates FcRn into the endosomal compartment at pH 6.0 and then induces the clearance of plasmatic antibodies, including pathogenic autoantibodies. Combined, these results indicate that LFBD192 represents an innovative recombinant molecule with potential for application in the treatment of IgG-dependent autoimmune pathologies.

## Data Availability Statement

The original contributions presented in the study are included in the article/supplementary material. Further inquiries can be directed to the corresponding author.

## Ethics Statement

All CAIA experiments were associated with the Project No. 17_045 submitted to the Ethics Committee in Animal Experimentation (EAEC) No. 44 and approved by “le ministère de l’éducation nationale, de l’enseignement supérieur et de la recherche” under the number APAFIS # 9410-201703271734338. All K/BxN experiments were conducted in Artimmune laboratory (Orléans, France) and performed in compliance with the guidelines of the Agriculture French Ministry for experiments with laboratory animals (law 87-848). All animal experiments were approved by the “Ethics Committee for Animal Experimentation of CNRS Campus Orleans” (CCO) under number CLE CCO 2015-1081.

## Author Contributions

CM designed the mutants and analyzed the data. AB and AT produced the variants in HEK cells and characterized them. EJ wrote and revised the manuscript, designed and analyzed all the *in vivo* experiments, and performed the *in vivo* CAIA experiments. GD, DD, and AE performed some of the *in vitro* experiments. CRo and NF designed and analyzed some of the *in vitro* experiments. CB and AL performed production, purification of LFBD192 at a larger scale, and characterizations. AF performed the *in-silico* immunogenicity study and with TA, designed and analyzed production, purification at a larger scale of LFBD192 and revised the manuscript. AS performed and analyzed the Biacore experiments. LD, MN, and NM designed and performed LFBD192 CHO production and purification. AR and FD designed and performed the experiment involving the inhibition of antibody-mediated platelet phagocytosis. DT and LF designed and performed the K/BxN-related *in vivo* experiments. IS and FN designed and performed the *in vivo* ITP-related experiments. WS and SC designed the research and revised the manuscript. PM wrote the manuscript, designed and analyzed all the *in vivo* experiments, designed the research, and analyzed the data. All authors contributed to the article and approved the submitted version.

## Funding

The authors declare that this study received funding from LFB Biotechnologies, Les Ulis, France. The funder was not involved in the study design, collection, analysis, interpretation of data, the writing of this article or the decision to submit it for publication. FN is supported by grants from the German Research foundation (DFG-FOR2886 and CRC1181-A07).

## Conflict of Interest

Authors CM, EJ, CRe, AF, TA, NF, GD, DD, AE, AB, AT, AS, CB, AL, NM, LD, MN, AR, FD, WS, SC and PM are employees of LFB Biotechnologies, which patented the described Fc mutations. DT and LF are employees of Artimmune.

The remaining authors declare that the research was conducted in the absence of any commercial or financial relationships that could be construed as a potential conflict of interest.

## Publisher’s Note

All claims expressed in this article are solely those of the authors and do not necessarily represent those of their affiliated organizations, or those of the publisher, the editors and the reviewers. Any product that may be evaluated in this article, or claim that may be made by its manufacturer, is not guaranteed or endorsed by the publisher.
